# High Gama-Aminobutyric Acid Contents Involved in Abamectin Resistance and Predation, an Interesting Phenomenon in Spider Mites

**DOI:** 10.3389/fphys.2017.00216

**Published:** 2017-04-11

**Authors:** Zhifeng Xu, Yanchao Liu, Peng Wei, Kaiyang Feng, Jinzhi Niu, Guangmao Shen, Wencai Lu, Wei Xiao, Jinjun Wang, Guy J. Smagghe, Qiang Xu, Lin He

**Affiliations:** ^1^Key Laboratory of Entomology and Pest Control Engineering of Chongqing, College of Plant Protection, Southwest UniversityChongqing, China; ^2^Department of Crop Protection, Faculty of Bioscience Engineering, Ghent UniversityGhent, Belgium; ^3^Department of Biology, Abilene Christian UniversityAbilene, TX, USA

**Keywords:** *Tetranychus cinnabarinus*, abamectin, resistance, GABA, fitness cost, predator

## Abstract

Abamectin has been widely used as an insecticide/acaricide for more than 30 years because of its superior bioactivity. Recently, an interesting phenomenon was identified in the carmine spider mite, *Tetranychus cinnabarinus*, an important pest in agriculture. The gamma aminobutyric acid (GABA) contents in a laboratory abamectin resistant strain of *T. cinnabarinus* (AbR) were significantly increased. Decreases in activity and mRNA expression of GABA transaminase (GABA-T) were responsible for GABA accumulation in AbR mites. To clarify the mechanism of GABA accumulation mediated abamectin resistance, three artificial approaches were conducted to increase GABA contents in susceptible mites, including feeding of vigabatrin (a specific inhibitor of GABA-T), feeding of exogenous GABA, and inhibition of GABA-T gene expression. The results showed that susceptible mites developed resistance to abamectin when the GABA contents were artificially increased. We also observed that the mites with higher GABA contents moved more slowly, which is consistent with the fact that GABA is an inhibitory neurotransmitter in arthropods. Subsequently, functional response assays revealed that predation rates of predatory mites on GABA accumulated abamectin-resistant mites were much higher than control groups. The tolerance to abamectin, slow crawling speed, and vulnerability to predators were all resulted from GABA accumulation. This relationship between GABA and predation was also confirmed in a field-collected population. Our finding indicates that predatory mites might be used as a tool for biological control to circumvent the development of abamectin resistance in mites.

## Introduction

Spider mites have been one of the most polyphagous arthropods on this planet. They feed on more than 1,100 different plants in the field or in green houses (Grbić et al., 2011). The control of spider mites has been performed almost exclusively with the application of acaricides (Watanabe et al., 1994). However, the intensive use of these products has reduced the efficacy of the main active ingredients available on the market due to the rapid evolution of resistance in mite pests (Nauen et al., 2001; Van Leeuwen et al., 2010). An alternative method for the management of spider mites is biological control with predatory mites, which are considered effective natural enemies of phytophagous mites (Helle and Sabelis, 1985). However, the use of acaricides and predatory mites is mutually exclusive in most cases due to the fact that most acaricides are lack of selectivity between phytophagous and predatory mites.

The carmine spider mite (CSM), *Tetranychus cinnabarinus* (Boisduval) (Acarina: Tetranychidae), is widely distributed around the world. For many years, the control of *T. cinnabarinus* has traditionally relied on sprays of acaricides and control has been reported (Guo et al., 1998). Avermectins were discovered from secondary metabolites of the cosmopolitan soil bacterium *Streptomyces avermitilis* (McKellar and Benchaoui, 1996) and are very effective in controlling infections of parasites roundworm and as novel therapy against Malaria. This work was also awarded the 2015 Nobel Prize in Physiology or Medicine. Macrocyclic lactone abamectin (a mixture of avermectin B1a and B1b) has been recognized globally as a successful example of pesticide commercialization from microbiological origin (Holden-Dye and Walker, 1990; Lasota and Dybas, 1991). It has a broad spectrum of activities against arthropods, including insects and mites (Argentine and Clark, 1990; Strong, 1992). However, its extensive use over many years has led to the development of resistance in several insect pests (Rousw and Wright, 1986; Liang et al., 2003; Xin et al., 2010). A survey of pesticide resistance of *T. cinnabarinus* in Southern China revealed that field strains had different levels of resistance to three acaricides, including fenproathrin, omethoate, and propargite (Chen et al., 2012). However, these field populations were still relatively susceptible to abamectin (Chen et al., 2012). Resistance against abamectin in arthropods and nematodes is thought to be dependent on the expression changes of a diverse set of proteins, including glutamate gated chloride channels (GluCls), P-glycoproteins, and metabolic enzymes (Konanz and Nauen, 2004; Kwon et al., 2010; Dermauw et al., 2012; Luo et al., 2013; Riga et al., 2014). Some other interesting gamma-aminobutyric acid (GABA)-dependent phenomena were also found to be involved in abamectin toxicity. It is believed that abamectin works by activating GABA transporters, which stimulates pre-synaptic neurons to release excessive GABA, causing a prolonged GABAergic effect in post-synaptic neurons (Buckingham et al., 2006). This produces a highly specific and intense inhibitory effect, leading to death via anesthesia and thus achieving the desired pesticide effect (Lasota and Dybas, 1991).

GABA, widely present in most prokaryotic and eukaryotic organisms (Lee et al., 2011), is a major inhibitory neurotransmitter in the central nervous system (CNS) of vertebrates and various invertebrates including insects and acarids (Krnjević, 2004; Enell et al., 2007; Schousboe and Waagepetersen, 2007; Abbas et al., 2014). The cellular GABA level reflects a dynamic balance between synthesis and catabolism and is determined by the relative fluxes generated by two pyridoxal-5′-phosphate dependent enzyme, glutamate decarboxylase (GAD; EC 4.1.1.15) and GABA transaminase (GABA-T; EC 2.6.1.19) (de Graaf et al., 2006; Patel et al., 2006). GABA is mainly synthesized from L-glutamate in the CNS. Its synthesis takes place primarily in GABAergic neurons due to the activity of the cytosolic GAD. GABA can be transported into astrocytes to be catabolized by the enzyme GABA-T, which converts GABA to succinic semialdehyde (SSA). Several recent studies have shown that the immune system is capable of synthesizing and releasing the classical neurotransmitter GABA (Jin et al., 2013). As an inhibitory neurotransmitter, GABA has been widely applied to treat a variety of neurological diseases via increasing its level in CNS, such as epilepsy and Parkinson's disease (McGeer and McGeer, 1976; Stelzer et al., 1987).

High levels of GABA in abamectin-resistant mites were documented in one of our previous studies (Xin-jun et al., 2010), however the mechanism underlying the GABA accumulation in abamectin-resistant mites was not clear then. GAD and GABA-T, as pacing factors for GABA balance in organisms, may play key roles of GABA accumulation in abamectin resistant mites. Therefore, in this research, we investigated the divergence of GAD and GABA-T between susceptible and resistant mites to study the mechanism of GABA accumulation. Our current research also aimed to verify that if GABA accumulation was involved in abamectin resistance and to evaluate fitness cost in abamectin-resistant mites with a high GABA-level.

## Materials and methods

### Mite strains

The susceptible strain (SS) was collected from the fields of Beibei District, Chongqing, China in 1998, and was transferred to fresh cowpea seedlings without pesticide treatments. The abamectin resistant strain (AbR) was continuously selected from SS with abamectin in the laboratory and has about 30-folds of resistance level compared with the SS strain. The fenpropathrin resistant strain (FeR) was also selected from SS with fenpropathrin continuously and has about 100-folds of resistance level. The FeR was used as a control resistant strain in this study. Wild strain (WS), a field population with 2.5-fold of resistance level, was collected from the fields of Beibei, Chongqing, in 2015.The rearing conditions of all strains were: 26 ± 1°C temperature, 35–55% humidity, and 14:10 (L: D) photoperiod.

### Treatments

The leaf disk method was used in chemical treatment. Water was added to a glass culture dish with a diameter of 9 cm, and a sponge (3 × 3 × 2 cm) was placed in each dish. The sponge was wetted by water and covered by a piece of filter paper. Smooth cowpea leaves were put on the wet filter paper with their back facing up. Three-day old healthy adult females were gently transferred to the leaves and placed for 2 h to make them stable. Potter spray tower (Rothamsted, UK) was used to spray desired concentrations of abamectin, GABA or vigabatrin on the SS, and the treated mites were recorded as SS-A, SS-G and SS-V, respectively.

### Toxicity bioassays

Toxicity bioassays were measured with modified residual coated vial (RCV) method (Xin-jun et al., 2010). Five different concentrations of abamectin were poured into 2 mL centrifuge tubes. The centrifuge tubes were incubated with abamectin for 30 min, and then the chemicals were abandoned. The tubes were left for air dry. Thirty 3–5 days old adult females were put into each individual centrifuge tube and the survival rates were recorded under anatomical microscope after 24 h. Mites that did not move or irregularly trembled their legs were considered dead. Three replicates were conducted, and each replicate contained 30 mites.

### Crawling speed measurement

Healthy adult females (3-day-old) from different strains and different treatments were placed into the middle of a glass rod (10 cm long and 0.5 mm diameter). The time that the mite crawled from the mid-point to either end of the rod continuously and straightly was recorded to calculate the crawling speed. One hundred mites were measured in each strain or treatment group.

### Preference of predator on different mites

The selection frequencies of predatory mites were measured by a four-side device. The cross style sponge, covered by filter paper in the same size, was placed into 6cm-diameter-dish and soaked in water. A circular black light paper with 2 cm diameter was placed in the central part and four circular leaves with 2 cm diameter were placed in four directions 2 mm apart from the center. Mites from different strains or treatments were placed on three directions of the device, respectively, and the remaining direction was set as blank control. A 24-h starved predatory mite, *Neoseiulus barkeri* (Hughes), was placed in the middle. When it crawled across the water to certain leaf was regarded as a choice. Three replicates with at least 30 predatory mites were conducted.

### Attack ability test of the predatory mite

According to McMurtry and Scriven (1964), the sponge (3 cm diameter) was placed into dish (6 cm diameter), covered by filter paper in same size, and soaked in water. A circular leaf (2 cm diameter) was placed on the filter paper. The number of CSMs that put on each leaf was set as 3, 6, 9, 12, 15, or 18, respectively. A starved predatory mite was then transferred to each leaf. The result would be recorded 24 h later. Each strain and treatment group was measured for three times. The data was handled with the model of Hooling II: Na = aTN/(1+aThN). N—density of prey; Na—number of prey eaten during a period of time searching; a—attack rate or searching efficiency; T—total time spend; Th—handling time. The value of a/Th was used to value the predacious ability, or the rate of the carmine spider was attacked (Agiza et al., 2009).

### Measurement of GABA contents

The chromatographic conditions used for this study were as follows: A Hitachi L-2000 HPLC system (UV detector) and an Alltima C18 reverse phase column were utilized. Column temperature was 40°C with UV at wave length of 254 nm. Mobile phase A: acetonitrile (70 mmol/L)/acetic acid buffer (pH 6.5) = 25/975 (v/v); mobile phase B: acetonitrile/water/methanol = 450/400/150 (v/v); washing conditions A/B = 50/50 (v/v); flow rate was 0.8 mL/min and washing time was 15 min. Standard GABA samples and sample extracts (100 μL) were injected into test tubes. Drying agents (methanol/sodium acetate (1.0 mol/L)/triethylamine = 2/2/1 (v/v)) and 50μL of freeze-dried derivatizing agent (4-chlorophenylisothiocyanate/methanol/ethanol/triethylamine/water = 1/6/1/1/1 (v/v)) were also added. The derivatizing reaction occurred at room temperature for 30 min. The solution was freeze-dried and stored at 4°C. The sample was dissolved with 1mL diluting solution (5 mmol/L disodium hydrogen phosphate buffer, pH 7.4), and 10 μL of this sample was used for GABA content assessment. The diluting buffer was used as negative control. The entire experiment was replicated three times with three determinations per sample.

### Enzymatic assays

Two hundred adult females were homogenized in 1 mL PBS (0.05 mol/L pH7.0) on ice, then centrifuged at 10,000 g for 10 min at 4°C. The supernatant was used for testing. Protein concentration was measured by Coomassie brilliant blue method (Bradford, 1976). Using L-glutamate as substrate, enzyme solution and PBS were added to the reaction for 10 min incubation at 37°C.Then the colorant (6% redistilled phenol/5.2% sodium hypochlorite = 1/1) was mixed with reaction liquid for 20 min at 37°C. The reaction was measured at 630 nm using microplate reader (BioTek, USA).

To test the activity of GABA-T, γ-aminobutyrate and α-ketoglutarate (1:1) were used as substrates for 15 min incubation at 30°C. Enzyme solution and sodium phosphate (NAD^+^ 10-3 mol/L, pH8.75) were then added to the reaction for 60 min incubation at 30°C. The reaction was measured at 340 nm using microplate reader. The experiments were repeated for three times.

### Quantitative PCR

The 3-day-old adult females from SS, FeR, AbR, or WS strains were used for total RNA extraction and single strain cDNA was synthesized using PrimeScript RT reagent kit with gDNA eraser (TakaRa, Dalian, China). *RPS18* was used as a reference gene according to the expression stability evaluation (Sun et al., 2010). Primers used for qPCR are presented in Table S1. The qPCR was performed on Mx3000P thermocycler (Stratagene, USA). Quantification of the transcript level was calculated using the 2^−ΔΔCt^ method (Pfaffl, 2001). The qPCR experiments were repeated three times.

### RNAi of GABA-T genes

Since the nucleotide sequences of GABA-T^TC1^ and GABA-T^TC2^ were highly homologous, primers (GABA-T-RNAi) with T7 promoter was designed to amplify a common region of GABA-T^TC1^ and GABA-T^TC2^, and dsRNA was synthetized using TranscriptAid T7 High Yield Transcription Kit (Thermo Scientific, American). Primers (GABA-T-qPCR) used in qPCR were outside the dsRNA region for detection of RNAi efficiency of both GABA-T^TC1^ and GABA-T^TC2^. GFP dsRNAs were used as controls. Primer information was presented in Table S2.

Leaf-discs containing *dsRNA-GABA-T* to knock down the expression of GABA-T genes in *T. cinnabarinus* were prepared as follows: cowpea leaves were cut to a 1.5 cm diameter feeding arena, incubated at 60°C for 3 min for dehydration, and then separately treated with DEPC-water, GFP dsRNA, and *dsRNA-GABA-T* (10 μg/per leaf) for 5 h. After fully absorbed, the leaves were put on wet filter paper. Thirty female adults (3–5 d old and starved for 24 h) were placed in each leaf-disc. After feeding for 48 h, the mites were collected for the subsequent experiments.

## Results

### GABA accumulation in the AbR strain resulted from decreased expression of GABA-T

**Figure 1:**
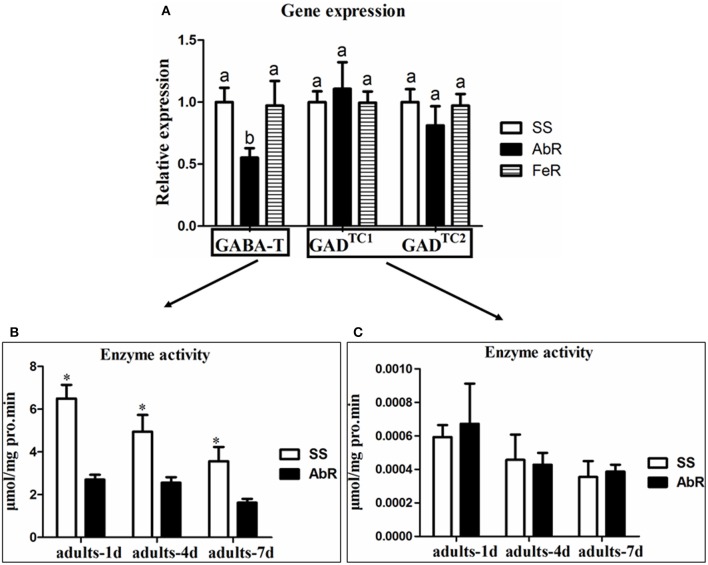
**The mechanism of GABA accumulation in AbR strain. (A)** Relative mRNA expression of GABA-T and GAD genes in different strains. **(B)** GABA-T enzyme assay in SS and AbR strains. **(C)** GAD enzyme assay in SS and AbR strains. Error bars indicate SE (*n* = 3). Asterisks and letters indicate a significant difference (*P* < 0.05).

Based on the CSM transcriptome data, two GAD and two GABA-T genes were cloned. According to the phylogenetic trees, the GAD and GABA-T genes in CSM showed the highest similarity with *T. urticae* (Figures S1, S2). Since GAD and GABA-T genes are two key enzymes in controlling GABA contents, their gene expression profiles in different mite strains were first investigated. The quantitative real time PCR results showed that the expression of GAD (GAD^TC1^ and GAD^TC2^) genes had no significant difference among susceptible (SS), fenpropathrin-resistant (FeR, used as extra control) and abamectin-resistant strains (AbR) (Figure 1A). However, the expression of GABA-T genes in AbR was significantly lower than that in SS or FeR (Figure 1A).

The enzyme activities of GAD and GABA-T, which catalyze the synthesis and catabolism of GABA, respectively, were also compared between SS and AbR. In the adult (adults-1, 4, and 7 d), GABA-T activity in AbR was significantly lower than that in SS (Figure 1B). However, there was no significant difference of GAD activity between SS and AbR (Figure 1C).

### Inhibition and silencing of GABA-T increased the content of GABA in CSM

In order to study the relationship between GABA accumulation and abamectin resistance in *T. cinnabarinus*, vigabatrin, a specific inhibitor of GABA-T, was used to treat the mites. The susceptible CSMs of SS strain were used to perform experiments since they have not developed GABA related resistance to abamectin. The activity of GABA-T could be inhibited *in vivo* by vigabatrin (0.443 mM) and 22.76% of activity was inhibited after susceptible CSMs were exposed with vigabatrin for 4 h (Figure S3).

**Figure 2:**
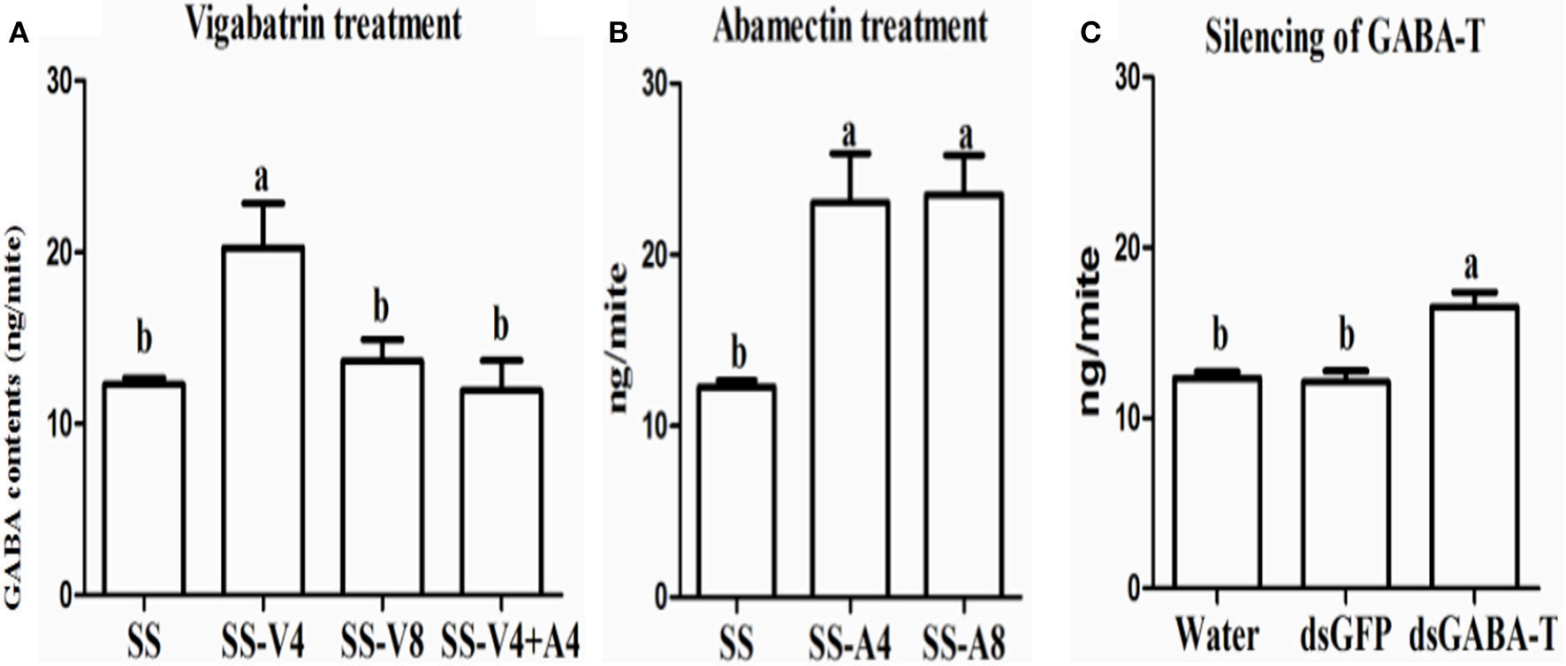
**GABA contents among treatments in the susceptible SS strain. (A)** Vigabatrin treatment. **(B)** Abamectin treatment. **(C)** RNAi of GABA-T genes. Error bars indicate SE (*n* = 3). Letters indicate a significant difference (*P* < 0.05).

The results of high performance liquid chromatography (HPLC) showed that the GABA level in mites increased about 2-times after treating with vigabatrin (0.443 mM for 4 h) *in vivo* compared with the control (Figure 2A, SS-V4 *VS*. SS). However, the GABA returned to almost the same level 8 h after the treatment (Figure 2A, SS-V8), indicating that vigabatrin (0.443 mM) did increase the GABA content in CSM *in vivo* and the effect would not last for 8 h. The GABA content in SS (SS-A4) also increased about 2-times after sprayed with abamectin (6.25 μM) after 4 h and maintained in the high level after 8 h (SS-A8), suggesting that abamectin could stimulate GABA release in mites for more than 8 h (Figure 2B). However, when the GABA content was pre-induced to a higher level by vigabatrin, the treatment of abamectin could not increase the content of GABA anymore (Figure 2A, SS-V4+A4). The silencing of two GABA-T genes through RNA interference (RNAi) showed that the expression of GABA-T genes decreased about 25% after feeding with *dsRNA-GABA-T* (Figure S4) and the GABA content increased significantly (Figure 2C).

### Increased GABA content led to reduction in toxicity of abamectin in CSM

**Figure 3:**
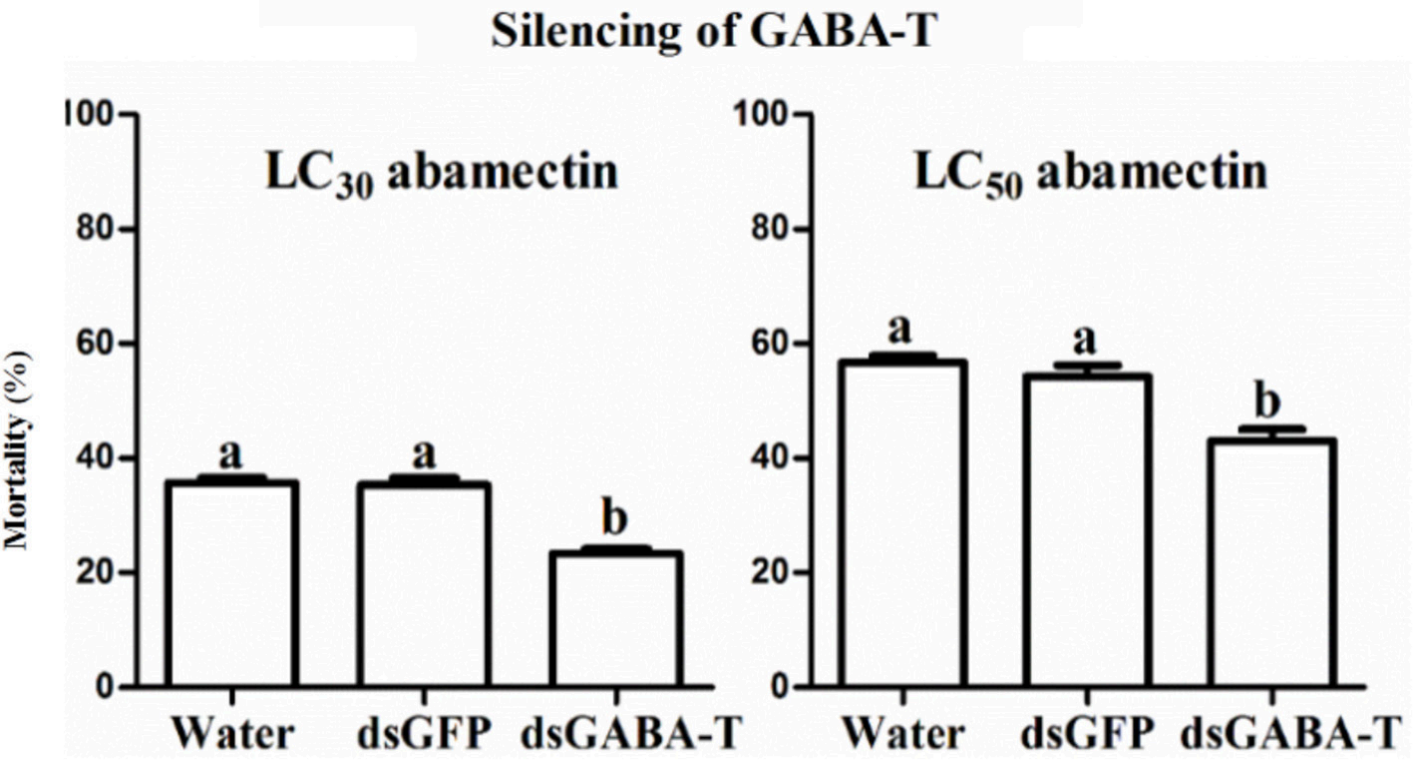
**The corrected mortality of SS mites when treated with LC_30_ and LC_50_ of abamectin after RNAi**. The significant difference (*P* < 0.05) was indicated by letters.

When the endogenous GABA was increased in the SS strain with vigabatrin (0.443 mM) treatment, the result of toxicity test showed that the resistance of SS against abamectin increased 4.1-fold (Table 1). A similar result was observed in the exogenous GABA-treated SS (SS-G), in which the mortalities were significantly lower compared with controls (SS) (Table 2). The effect of exogenous GABA weakened over time and when the doses of abamectin were increased (Table 2). For instance, when the applied dose of abamectin was 6.25 μM, the exogenous GABA treatment could help mites survive for more than 24 h before reaching to 100% mortality. However, when the doses of abamectin were increased to 25 or 100 μM, the exogenous GABA treatment could only help mites survive for 8 h before reaching to 100% mortality (Table 2). When the expression of GABA-T genes was suppressed via RNAi, the mortality of mites decreased about 10 and 15% with the exposure to LC_30_or LC_50_of abamectin, respectively (Figure 3). The results revealed that the toxicity of abamectin against the CSM could be partially reduced by an increase of the internal GABA level.

**Table 1:** **Toxicity of abamectin to the AbR strain and SS-V treatment compared with the susceptible SS strain**.

**Strains**	***N***	**Slope (±SE)**	***df***	**χ^2^**	**LC_50_(95%CL), mg/g**	**RR**
SS	495	2.8 (±0.4)	4	8.0	0.197 (0.168–0.252)	1
AbR	489	2.0 (±0.2)	4	4.3	5.471 (4.707–6.586)	27.8
SS-V	449	3.7 (±0.4)	4	18.8	0.801 (0.486–0.996)	4.1

*N, total number of trial mites; LC_50_, median lethal concentration; 95%CL, 95%confidence intervals; RR, resistance radio = LC_50_ (AbR or SS-V)/LC_50_ (SS); SS-V, the susceptible mites treated with vigabatrin (0.443 mM)*.

**Table 2:** **The mortality of SS-G and the SS strain after abamectin treatment**.

**Treatments**	**Corrected mortality** ± **95%CI (%)**		
	**4 h**	**8 h**	**24 h**
SS-G + 6.25 μM AVM	1.84 ± 0.85	4.05 ± 0.62	43.67 ± 20.03
SS + 6.25 μM AVM	4.2 ± 0.71^*^	43.67 ± 4.24^*^	79.88 ± 3.32^*^
SS-G + 25 μM AVM	12.61 ± 4.44	73.06 ± 11.34	100
SS + 25 μM AVM	43.35 ± 13.04^*^	100^*^	100
SS-G +100 μM AVM	25.59 ± 9.93	85.07 ± 10.67	100
SS + 100 μM AVM	57.28 ± 16.35^*^	100^*^	100

95% CI, 95% confidence intervals; SS-G, the susceptible mites treated with exogenetic GABA.

* *indicated significant difference between SS-G and SS when treated with abamectin in the same concentration*.

### The increased GABA content led to lower crawling speed of CSM

**Figure 4:**
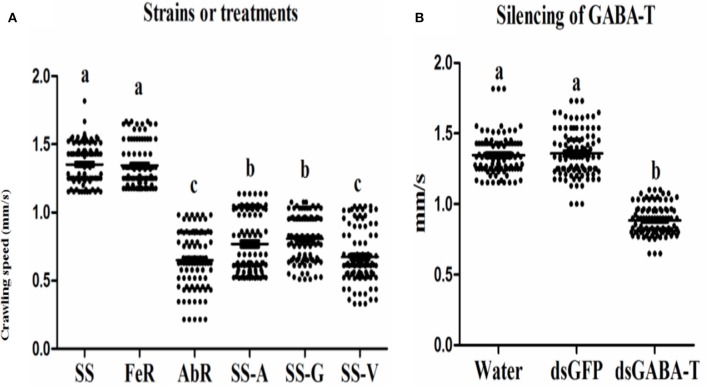
**Scatter plot of crawling speed results of different strains and treatments (*n* = 100)**. The significant difference (*P* < 0.05) was indicated by letters. **(A)** Indoor strains, **(B)** wild strain.

The measurement of crawling speed of CSMs in different treatments revealed that the AbR mites moved more slowly than SS and FeR (resistant strain control) mites, whereas there was no significant difference between SS and FeR mites (Figure 4A). When SS mites were treated with abamectin (6.25 μM), vigabatrin (0.443 mM), or exogenetic GABA (1 mM), the crawling speeds all decreased significantly compared with controls (Figure 4A). Meanwhile, through RNAi, the mites with lower expression of GABA-T moved more slowly compared with controls (Figure 4B), indicating that an increase of internal GABA levels resulted in the same physical response as the treatments of these three chemicals in mites.

### The higher predation rate by predatory mite was present in CSMs with higher GABA content

**Figure 5:**
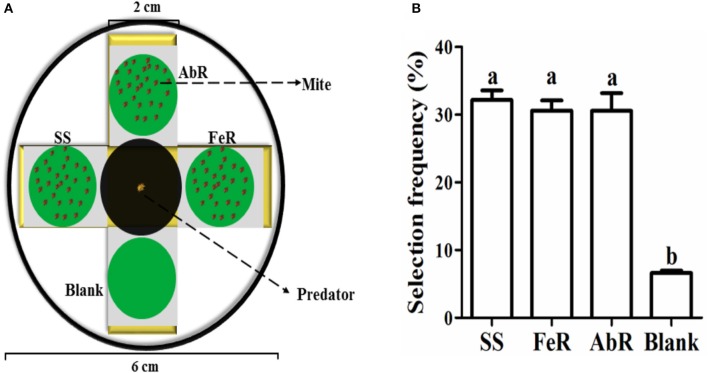
**Detection of predatory mite preference to pesticide susceptible and resistant mites. (A)** The device used for the selection frequency measurement of predatory mites. **(B)** The preferential choice of predatory mites to four directions. The significant difference (*P* < 0.05) was indicated by letters.

Mites from SS, AbR, and FeR strains were placed in three different directions of a four-side device (the rest direction was set as blank control) (Figure 5A). Then, a predatory mite was placed at the center, and its crawling direction was recorded to analyze the choice frequency. The results showed that predatory mites were more inclined to crawl toward to CSMs than the blank. However, its choice showed no preference among three strains (Figure 5B), which indicated that the predatory mite could locate the prey, but it had no preference between susceptible and resistant mites.

The mites from four different treatments, SS, AbR, SS-V, and SS-G, were, respectively, placed on the leaf disc located at the center of a petri dish (Figure S5). Subsequently, a predatory mite was placed on the leaf-disc to measure its attack ability according to Holling model (34). With a good fitness, the result indicated that SS-V, SS-G, and AbR mites were much easier to be captured by the predators than SS mites (a/Th: SS-V > SS-G > AbR > SS) (Table 3), indicating that high GABA contents made mites more difficult to escape from their predators. Interestingly, similar result was also achieved from RNAi experiments. The attack ability of predatory mites to CSMs fed with *dsRNA-GABA-T*, which possessed higher GABA contents, was significantly stronger than that to controls (fed with water or *dsRNA-GFP*) (Table 3).

**Table 3:** **The response of predacious function of predatory mite**.

**Strains (treatment)**	***a***	**Th (day)**	***a*/Th**	**Na = aTN/(1+aThN)**	***R*^2^**
**LABORATORY MITES**					
SS	0.64	0.27	2.36	Na = 0.64N/(1+0.17N)	0.86
AbR	0.84	0.23	3.75	Na = 0.84N/(1+0.19N)	0.84
SS-G	0.78	0.19	4.06	Na = 0.78N/(1+0.15N)	0.93
SS-V	1.29	0.25	5.17	Na = 1.29N/(1+0.32N)	0.90
**LABORATORY MITES AFTER RNAi**					
SS-CK	0.61	0.24	2.54	Na = 0.61N/(1+0.15N)	0.90
SS-dsGFP	0.62	0.29	2.17	Na = 0.62N/(1+0.18N)	0.73
SS-dsGABA-T	0.77	0.21	3.66	Na = 0.77N/(1+0.16N)	0.81
**FIELD MITES AFTER RNAi**					
WS	0.59	0.27	2.15	Na = 0.59N/(1+0.16N)	0.87
WS-CK	0.62	0.32	1.92	Na = 0.62N/(1+0.20N)	0.82
WS-dsGFP	0.63	0.34	1.87	Na = 0.63N/(1+0.21N)	0.84
WS-dsGABA-T	0.83	0.28	2.96	Na = 0.83N/(1+0.23N)	0.84

*a, Instantaneous attack rates; Th, disposal time; a/Th, predacious ability*.

*R^2^, determination coefficient*.

### A field strain also showed lower mobility and higher rate to be preyed on after GABA-T gene silencing

**Figure 6:**
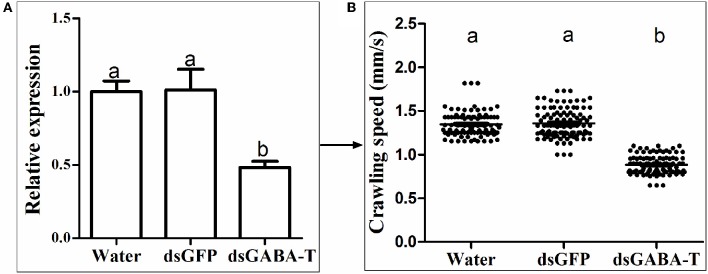
**The detection of GABA effect in a field strain (WS) via RNAi. (A)** Relative mRNA expression of GABA-T gene. The mRNA expression of GABA-T gene injected with water in SS strain as 1. **(B)** Crawling speed (*n* = 100). Error bars indicate SE (*n* = 3). The significant difference (*P* < 0.05) was indicated by letters.

To clarify if the phenomenon observed in our laboratory strains is also present in randomly collected field strains, a wild strain (WS) was collected to validate the GABA effect. This WS strain has 2.5-fold abamectin resistant ratio compared with the SS strain. Our results showed that there was no significant difference in GABA contents, GABA-T mRNA level and crawling speed between SS and WS strains (Figure S6). However, upon the silencing of the GABA-T genes by RNAi in the WS strains, the mites moved more slowly (Figure 6) and were consequently easier to be caught by predators (Table 3).

## Discussion

As the two most important enzymes in the GABAergic neurons, GAD is responsible for synthesis of GABA while GABA-T can metabolize GABA into SSA. In living organisms, either an increase of GAD activity or a decrease of GABA-T activity can result in the accumulation of GABA (Mazurkiewicz et al., 1999; de Graaf et al., 2006; Patel et al., 2006; Shimajiri et al., 2013). In this study, we aimed to clarify how GABA contents were increased in CSMs for the development of abamectin resistance and to study how GABA served as a pivot to balance the chemical (abamectin) and biological (predator) control of mites. The significant decreases of GABA-T gene expression and enzyme activity in abamectin-resistant mites were confirmed when compared with SS, while GAD expression showed no difference among different strains. These results suggested that the reduction of metabolic rate of GABA, due to the decrease of GABA-T activity, was the main reason for its accumulation in abamectin-resistant CSMs. A similar phenomenon was also found in mammals. A decrease of GABA-T activity also led to the accumulation of GABA in rats (de Graaf et al., 2006).

Three methods, such as spraying vigabatrin (a GABA-T specific inhibitor) to inhibit GABA-T's activity, supplying exogenous GABA to increase GABA contents and suppressing gene expression of GABA-T via RNAi, were adopted to elevate the GABA level artificially. Their effects on increasing GABA contents in mites were confirmed by detecting the amount of GABA with HPLC and testing behavior responses with crawling speed measurements. The results of bioassay revealed that these GABA-elevated mites became less sensitive to abamectin than controls, which leads to the question why the increase of GABA contents can cause abamectin resistance in mites? One mode of action of abamectin was reported as stimulating the release of GABA at the brain synaptosomes in rats (Pong et al., 1980). However, GABA is kept in a dynamic balance in organisms and high levels of GABA in synaptic cleft will also inhibit the GABA release from axon terminal vesicles, which is termed as the “negative feedback mechanism” (Mitchell and Martin, 1978; Otis and Mody, 1992; Mody et al., 1994). When the mites were exposed to abamectin, the individuals with high GABA contents could counteract the stimulation from abamectin for GABA release via negative feedback loop, which would decrease the acaricidal activity of abamectin. Thus, the CSM with high-level GABA developed resistance against abamectin. However, how abamectin-resistant mites can maintain normal nerve signal conduction in an environment with high of GABA contents remains to be an interesting topic for further investigation.

There are two main types of fitness costs of insecticide resistance correlated with two major resistant mechanisms. Fitness costs on biological traits (such as longer development period, lower fecundity, higher proportion of males, etc.) are more correlated with over-expression of detoxifying enzyme genes while fitness costs on behavioral traits (lower moving speed and lower responsive ability resulting in easier predation, parasitism and not easier avoiding hazardous environments) are more correlated with the insensitivity of target sites in the nervous system. Estimations of fitness costs from *Culex pipiens* population surveys revealed that *ace*-1 (acetylcholinesterase, a target for pesticides) was associated with higher deleterious effects than *Ester* (locus for two esterase enzymes) (Lenormand et al., 1999; Lenormand and Raymond, 2000). The over-production of esterase enzymes could be at the expense of the synthesis of other macromolecules resulting in costs on biological traits whereas the modified target, acetylcholinesterase, could lead to costs on behavioral traits since it alters the optimal functioning of cholinergic synapses of the central nervous system (Berticat et al., 2004).

**Figure 7:**
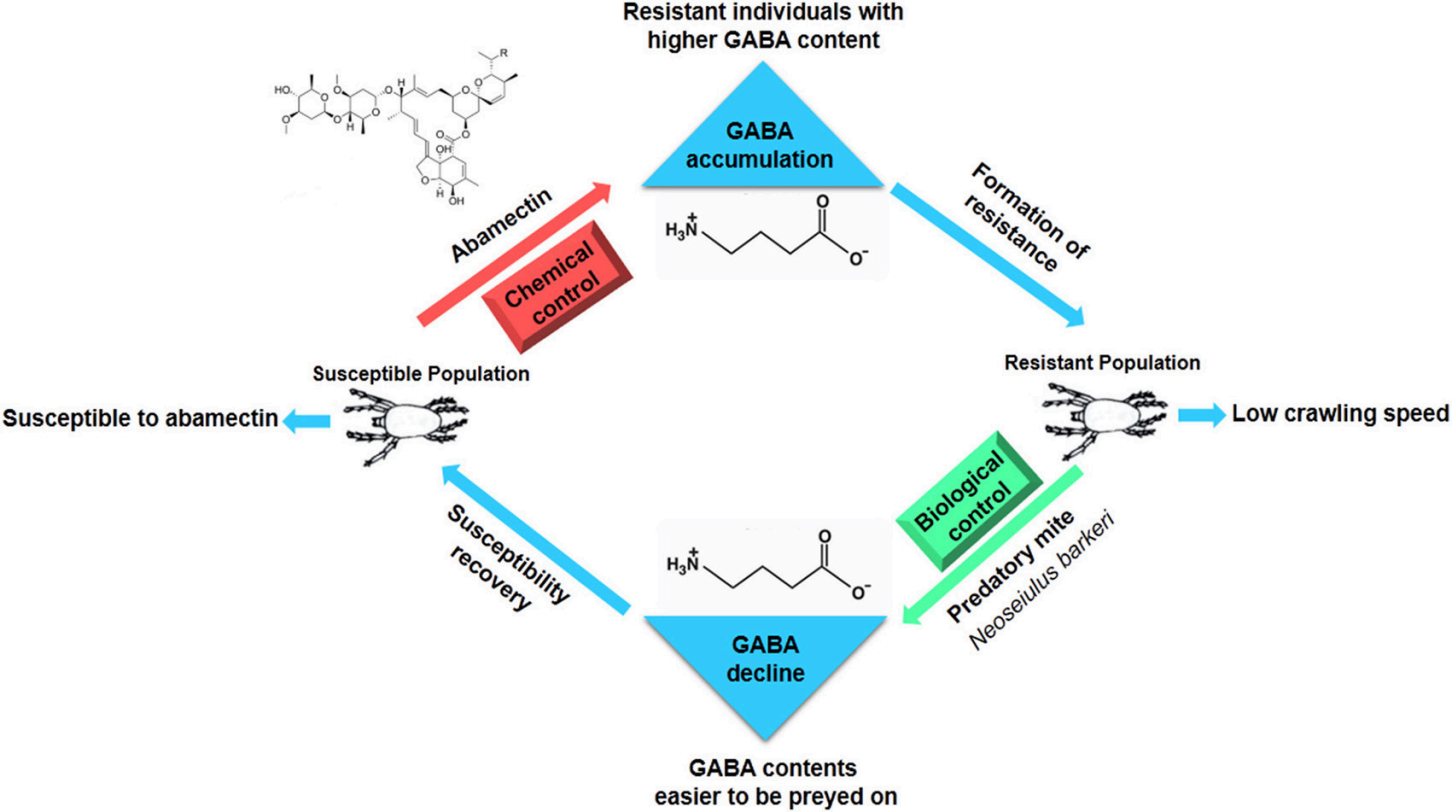
**GABA keeps the balance between chemical and biological control in *T. cinabarinus***.

Abamectin has been used as pesticides to control arthropods for more than 50 years and information regarding resistance monitoring and mechanisms has been accumulated over time. However, little is known on fitness costs of abamectin resistance. Wang and Wu found that abamectin resistance in *Plutella xylostella* had resulted in significant biological fitness costs (Wang and Wu, 2014). This study is a first report on behavioral fitness costs of abamectin-resistance. Results of crawling speed measurements revealed that not only that abamectin-resistant mites but also susceptible ones that were treated artificially to increase GABAcontents displayed a slower moving speed, suggesting that high level of GABA is the reason for the decrease in motor ability of *T. cinnabarinus* and the resistance against abamectin is at the expense of moving slowly. The functional response data of *Neoseiulus barkeri* (Hughes) on *T. cinnabarinus* indicated that the predation rates of *N. barkeri* on abamectin-resistant mites and susceptible individuals with an artificially increased GABA contents were much higher than that on susceptible mites. Similar to susceptible mites in the laboratory, when GABA-T activity of a field population was inhibited by RNAi, GABA contents in field mites and the predation rates of *N. barkeri* were higher than those in controls, indicating that the “GABA effect” exists not only in laboratory stains but also in wild population. Theoretically, predator' preference could affect the predation efficiency against different prey. The *N. barkeri* did show preference on leaf-disc with mites compared with empty control. However, the predatory mites showed no significant preference between acaricide-resistant and susceptible mites, from which the possibility of prey-selectivity preference contributing to higher predation rates can be excluded. Therefore, we conclude that the higher predation rates of *N. barkeri* on higher GABA-content mites attribute to the fact that these mites moved more slowly than individuals with normal GABA contents. The abamectin resistance in *T. cinnabarinus* confers a predation cost. The predation cost had also been documented in other pesticide resistant species. For instance, knock-down resistance to insecticides in *Myzus persicae* imposed a fitness cost through increased vulnerability to natural enemies (Foster et al., 1999). The presence of a resistance gene *(ace-1)* in *Culex pipiens* increased the probability of predation at both the larval and the adult stage (Berticat et al., 2004). The predation cost in abamectin-resistant *T. cinnabarinus* might provide a new interpretation why the resistance levels to abamectin were still relatively low in six field populations in Southern China (Chen et al., 2012) even though abamectin has been widely used to control arthropod pests for more than 30 years in China. More importantly, the present study sheds new light on balancing applications between the chemical and biological control (abamectin/predator) of *T. cinnabarinus*. When abamectin-resistance develops in field population, predatory mites can be released to control the resistant population and recover the susceptibility, thereby decreasing the pesticide usage and increasing the service lifespan of abamectin. GABA sits on a pivot position between chemical and biological control of CSMs (Figure 7).

In summary, we report here a very interesting phenomenon in abamectin resistance of spider mites. High gamma amino acid butyric acid (GABA) contents caused abamectin resistance in mites. On the other hand, high GABA contents also made mites more vulnerable to their natural enemies.

## Author contributions

LH, GJS, JN, and QX designed research; ZX, YL, PW, and WL performed research; ZX, YL, PW, GS, LH, and QX analyzed data; ZX, KF, GS, WX, and JN. JW, GJS, QX, and LH wrote the paper.

## Funding

This research was funded in part by the National Natural Science Foundation of China (31672085), the China Postdoctoral Science Foundation (2016M602638), the Fundamental Research Funds for the Central Universities (XDJK2016A005), and the Foundation Project of Southwest University (SWU114049). This work was also supported by Abilene Christian University Faculty Renewal Leave Grants, Pursuit Grants, and Math and Science Grants.

### Conflict of interest statement

The authors declare that the research was conducted in the absence of any commercial or financial relationships that could be construed as a potential conflict of interest. The reviewer JS and handling Editor declared their shared affiliation, and the handling Editor states that the process nevertheless met the standards of a fair and objective review.

## References

[B1] Abbas R. Z. Zaman M. A. Colwell D. D. Gilleard J. Iqbal Z. (2014). Acaricide resistance in cattle ticks and approaches to its management: the state of play. Vet. Parasitol. 203, 6–20. 10.1016/j.vetpar.2014.03.006 24709006

[B2] Agiza H. Elabbasy E. El-Metwally H. Elsadany A. (2009). Chaotic dynamics of a discrete prey–predator model with Holling type II. Nonlin. Anal. Real. 10, 116–129. 10.1016/j.nonrwa.2007.08.029

[B3] Argentine J. A. Clark J. M. (1990). Selection for abamectin resistance in Colorado potato beetle (Coleoptera: Chrysomelidae). Pestic Sci. 28, 17–24. 10.1002/ps.2780280104

[B4] Berticat C. Duron O. Heyse D. Raymond M. (2004). Insecticide resistance genes confer a predation cost on mosquitoes, *Culex pipiens*. Genet. Res. 83, 189–196. 10.1017/S0016672304006792 15462412

[B5] Bradford M. M. (1976). A rapid and sensitive method for the quantitation of microgram quantities of protein utilizing the principle of protein-dye binding. Anal. Biochem. 72, 248–254. 10.1016/0003-2697(76)90527-3 942051

[B6] Buckingham S. D. Biggin P. C. Sattelle B. M. Brown L. A. Sattelle D. B. (2006). Insect GABA receptors: splicing, editing, and targeting by antiparasitics and insecticides. Mol. Pharm. 68, 942–951. 10.1124/mol.105.015313 16027231

[B7] Chen Q. S. Zhao S. Shi L. Zou J. He L. (2012). Monitoring of acaricide resistance in *Tetranychus cinnabarinus*. Chin. J. Appl. Entomol. 2:10 10.7679/j.issn.2095-1353.2012.049

[B8] de Graaf R. A. Patel A. B. Rothman D. L. Behar K. L. (2006). Acute regulation of steady-state GABA levels following GABA-transaminase inhibition in rat cerebral cortex. Neurochem. Int. 48, 508–514. 10.1016/j.neuint.2005.12.024 16517019

[B9] Dermauw W. Ilias A. Riga M. Tsagkarakou A. Grbić M. Tirry L.. (2012). The cys-loop ligand-gated ion channel gene family of *Tetranychus urticae*: implications for acaricide toxicology and a novel mutation associated with abamectin resistance. Insect Biochem. Mol. Biol. 42, 455–465. 10.1016/j.ibmb.2012.03.002 22465149

[B10] Enell L. Hamasaka Y. Kolodziejczyk A. Nässel D. R. (2007). γ-Aminobutyric acid (GABA) signaling components in *Drosophila*: immunocy to chemical localization of GABAB receptors in relation to the GABAA receptor subunit RDL and a vesicular GABA transporter. J. Comp. Neurol. 505, 18–31. 10.1002/cne.21472 17729251

[B11] Foster S. P. Woodcock C. M. Williamson M. S. Devonshire A. L. Thompson I. D. R. (1999). Reduced alarm response by peach–potato aphids, *Myzus persicae* (Hemiptera: Aphididae), with knock-down resistance to insecticides (kdr) may impose a fitness cost through increased vulnerability to natural enemies. Bull. Entomol. Res. 89, 133–138. 10.1017/S0007485399000218

[B12] Grbić M. Van L. T. Clark R. M. Rombauts S. Rouzé P. Grbić V.. (2011). The genome of *Tetranychus urticae* reveals herbivorous pest adaptations. Nature 479, 487–492. 10.1038/nature10640 22113690 PMC4856440

[B13] Guo F. Zhang Z. Q. Zhao Z. (1998). Pesticide resistance of *Tetranychus cinnabarinus* (Acari: Tetranychidae) in China: a review. Syst. Appl. Acarol. 3, 3–7. 10.11158/saa.3.1.1

[B14] Helle W. Sabelis M. W. (1985). Spider Mites: Their Biology, Natural Enemies and Control, vol. 1 Amsterdam: Elsevier.

[B15] Holden-Dye L. Walker R. J. (1990). Avermectin and avermectin derivatives are antagonists at the 4-aminobutyric acid (GABA) receptor on the somatic muscle cells of Ascaris; is this the site of anthelmintic action? Parasitology 101:265. 2175874 10.1017/s0031182000063320

[B16] Jin Z. Mendu S. K. Birnir B. (2013). GABA is an effective immune modulatory molecule. Amino Acids 45, 87–94. 10.1007/s00726-011-1193-7 22160261 PMC3680704

[B17] Konanz S. Nauen R. (2004). Purification and partial characterization of a glutathione S-transferase from the two-spotted spider mite, *Tetranychus urticae*. Pestic Biochem. Phys. 79, 49–57. 10.1016/j.pestbp.2004.03.004

[B18] Krnjević K. (2004). How does a little acronym become a big transmitter? Biochem. Pharmacol. 68, 1549–1555. 10.1016/j.bcp.2004.06.038 15451398

[B19] Kwon D. H. Yoon K. S. Clark J. M. Lee S. H. (2010). A point mutation in a glutamate-gated chloride channel confers abamectin resistance in the two-spotted spider mite, *Tetranychus urticae* Koch. Insect. Mol. Biol. 19, 583–591. 10.1111/j.1365-2583.2010.01017.x 20522121

[B20] Lasota J. A. Dybas R. A. (1991). Avermectins, a novel class of compounds: implications for use in arthropod pest control. Annu. Rev. Entomol. 36, 91–117. 10.1146/annurev.en.36.010191.000515 2006872

[B21] Lee M. Mcgeer E. G. Mcgeer P. L. (2011). Mechanisms of GABA release from human astrocytes. Glia 59, 1600–1611. 10.1002/glia.21202 21748804

[B22] Lenormand T. Bourguet D. Guillemaud T. Raymond M. (1999). Tracking the evolution of insecticide resistance in the mosquito *Culex pipiens*. Nature 400, 861–864. 10.1038/23685 10476962

[B23] Lenormand T. Raymond M. (2000). Analysis of clines with variable selection and variable migration. Am. Nat. 155, 70–82. 10.1086/303295 10657178

[B24] Liang P. Gao X. W. Zheng B. Z. (2003). Genetic basis of resistance and studies on cross-resistance in a population of diamondback moth, *Plutella xylostella* (Lepidoptera: Plutellidae). Pest Manag. Sci. 59, 1232–1236. 10.1002/ps.760 14620050

[B25] Luo L. Sun Y. J. Yang L. Huang S. Wu Y. J. (2013). Avermectin induces P-glycoprotein expression in S2 cells via the calcium/calmodulin/NF-κ B pathway. Chem. Biol. Interact. 203, 430–439. 10.1016/j.cbi.2013.03.009 23523950

[B26] Mazurkiewicz M. Opolski A. Wietrzyk J. Radzikowski C. Kleinrok Z. (1999). GABA level and GAD activity in human and mouse normal and neoplastic mammary gland. J. Exp. Clin. Cancer Res. 18, 247–253. 10464715

[B27] McGeer P. L. McGeer E. G. (1976). Enzymes associated with the metabolism of catecholamines, acetylcholine and GABA in human controls and patients with Parkinson's disease and Huntington's chorea. J. Neurochem. 26, 65–76. 3629

[B28] McKellar Q. A. Benchaoui H. A. (1996). Avermectins and milbemycins. J. Vet. Pharmacol. Ther. 19, 331–351. 10.1111/j.1365-2885.1996.tb00062.x 8905567

[B29] McMurtry J. A. Scriven G. T. (1964). Studies on the feeding, reproduction, and development of *Amblyseius hibisc*i (Acarina: Phytoseiidae) on various food substances. Ann. Entomol. Soc. Am. 57, 649–655. 10.1093/aesa/57.5.649

[B30] Mitchell P. R. Martin I. L. (1978). Is GABA release modulated by presynaptic receptors? Nature 274, 904–905. 210397 10.1038/274904a0

[B31] Mody I. De Koninck Y. Otis T. S. Soltesz I. (1994). Bridging the cleft at GABA synapses in the brain. Trends Neurosci. 17, 517–525. 10.1016/0166-2236(94)90155-4 7532336

[B32] Nauen R. Stumpf N. Elbert A. Zebitz C. P. Kraus W. (2001). Acaricide toxicity and resistance in larvae of different strains of *Tetranychus urticae* and *Panonychus ulmi* (Acari: Tetranychidae). Pest Manag. Sci. 57, 253–261. 10.1002/ps.280 11455655

[B33] Otis T. S. Mody I. (1992). Modulation of decay kinetics and frequency of GABA_*A*_ receptor-mediated spontaneous inhibitory postsynaptic currents in hippocampal neurons. Neuroscience 49, 13–32. 10.1016/0306-4522(92)90073-B 1357584

[B34] Patel A. B. de Graaf R. A. Martin D. L. Battaglioli G. Behar K. L. (2006). Evidence that GAD65 mediates increased GABA synthesis during intense neuronal activity *in vivo*. J. Neurochem. 97, 385–396. 10.1111/j.1471-4159.2006.03741.x 16539672

[B35] Pfaffl M. W. (2001). A new mathematical model for relative quantification in real-time RT–PCR. Nucleic Acids Res. 29:e45. 10.1093/nar/29.9.e45 11328886 PMC55695

[B36] Pong S. S. Wang C. C. Fritz L. C. (1980). Studies on the mechanism of action of avermectin B_1_a: stimulation of release of γ-aminobutyric acid from brain synaptosomes. J. Neurochem. 34, 351–358. 10.1111/j.1471-4159.1980.tb06604.x 7411150

[B37] Riga M. Tsakireli D. Ilias A. Morou E. Myridakis A. Stephanou E. G.. (2014). Abamectin is metabolized by CYP392A16, a cytochrome P450 associated with high levels of acaricide resistance in *Tetranychus urticae*. Insect Biochem. Mol. Biol. 46, 43–53. 10.1016/j.ibmb.2014.01.006 24463358

[B38] Rousw R. T. Wright J. E. (1986). Abamectin: toxicity to house flies (Diptera: Muscidae) resistant to synthetic organic insecticides. J. Econ. Entomol. 79, 562–564. 10.1093/jee/79.3.562 3722588

[B39] Schousboe A. Waagepetersen H. S. (2007). GABA: homeostatic and pharmacological aspects. Prog. Brain Res. 160, 9–19. 10.1016/S0079-6123(06)60002-2 17499106

[B40] Shimajiri Y. Oonishi T. Ozaki K. Kainou K. Akama K. (2013). Genetic manipulation of the γ-aminobutyric acid (GABA) shunt in rice: overexpression of truncated glutamate decarboxylase (GAD2) and knockdown of γ-aminobutyric acid transaminase (GABA-T) lead to sustained and high levels of GABA accumulation in rice kernels. Plant Biotechnol. J. 11, 594–604. 10.1111/pbi.12050 23421475

[B41] Stelzer A. Slater N. T. ten Bruggencate G. (1987). Activation of NMDA receptors blocks GABAergic inhibition in an *in vitro* model of epilepsy. Nature 326, 698–701. 10.1038/326698a0 2882427

[B42] Strong L. (1992). Avermectins: a review of their impact on insects of cattle dung. Bull. Entomol. Res. 82, 265 10.1017/S0007485300051816

[B43] Sun W. Jin Y. He L. Lu W. C. Li M. (2010). Suitable reference gene selection for different strains and developmental stages of the carmine spider mite, *Tetranychus cinnabarinus*, using quantitative real-time PCR. J. Insect Sci. 10, 208–208. 10.1673/031.010.20801 21265619 PMC3029232

[B44] Van Leeuwen T. Vontas J. Tsagkarakou A. Dermauw W. Tirry L. (2010). Acaricide resistance mechanisms in the two-spotted spider mite *Tetranychus urticae*, and other important acari: a review. Insect Biochem. Mol. Biol. 40, 563–572. 10.1016/j.ibmb.2010.05.008 20685616

[B45] Wang R. Wu Y. (2014). Dominant fitness costs of abamectin resistance in *Plutella xylostella*. Pest Manag. Sci. 70, 1872–1876. 10.1002/ps.3741 24464854

[B46] Watanabe M. A. Moraes G. J. D. Gastaldo I. Nicolella G. (1994). Biological control of the two-spotted spider mite (Acari: Tetranychidae, Phytoseiidae) in cucumber and strawberry crops. Sci. Agr. 51, 75–81. 10.1590/S0103-90161994000100012

[B47] Xin P. Yang Y. H. Wu S. W. Wu Y. D. (2010). Characterization of abamectin resistance in a field-evolved multi resistant population of *Plutella xylostella*. Pest Manag. Sci. 66, 371–378. 10.1002/ps.1885 19937910

[B48] Xin-jun Z. Wen-cai L. Ya-ning F. Lin H. (2010). High γ-aminobutyric acid content, a novel component associated with resistance to abamectin in *Tetranychus cinnabarinus* (Boisduval). J. Insect Physiol. 56, 1895–1900. 10.1016/j.jinsphys.2010.08.011 20713058

