# Supplementary figures and images for: High Gama-Aminobutyric Acid Contents Involved in Abamectin Resistance and Predation, an Interesting Phenomenon in Spider Mites
Source: Front Physiol. 2017 Apr 11;8:216. doi: 10.3389/fphys.2017.00216 (PMC5387048; doi:10.3389/fphys.2017.00216)

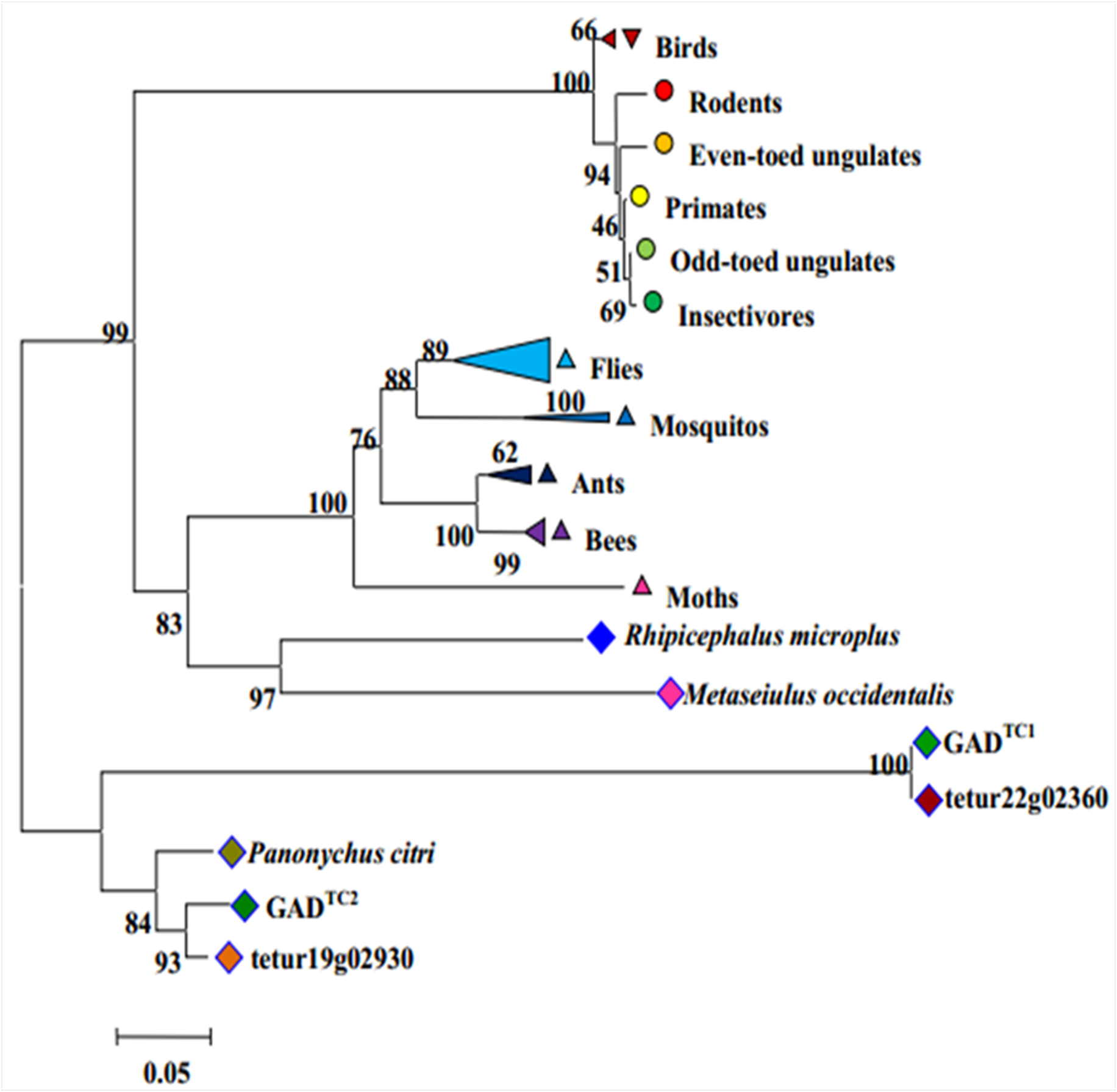

Supplement: Supplementary file 2 [file Presentation2.ZIP › Supplemetary Figures/Figure S1.tif]

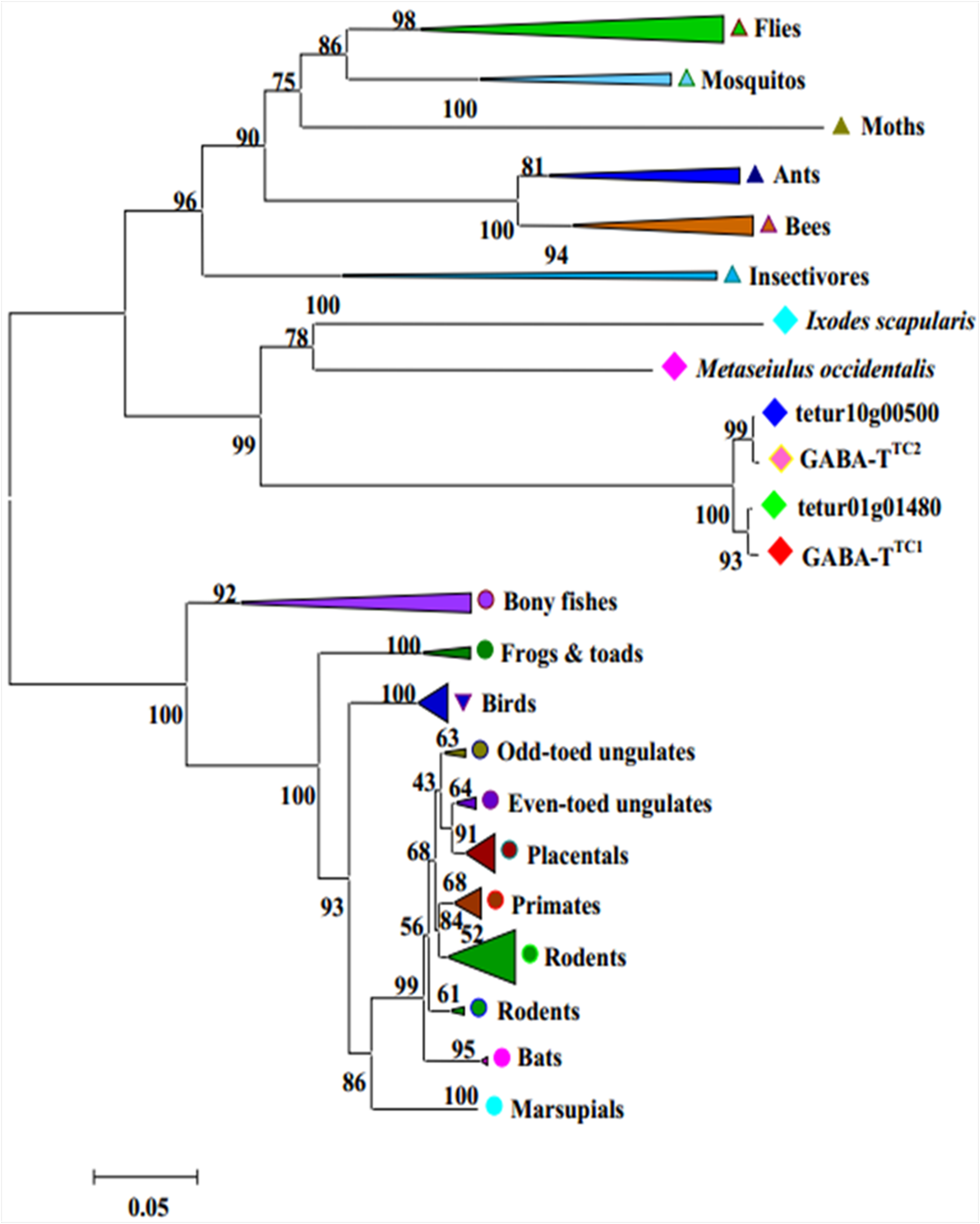

Supplement: Supplementary file 2 [file Presentation2.ZIP › Supplemetary Figures/Figure S2.tif]

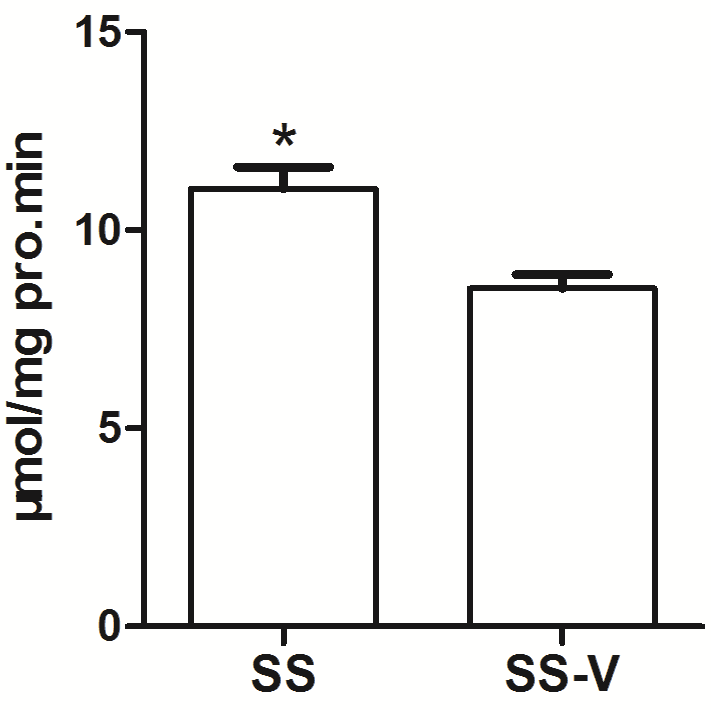

Supplement: Supplementary file 2 [file Presentation2.ZIP › Supplemetary Figures/Figure S3.tif]

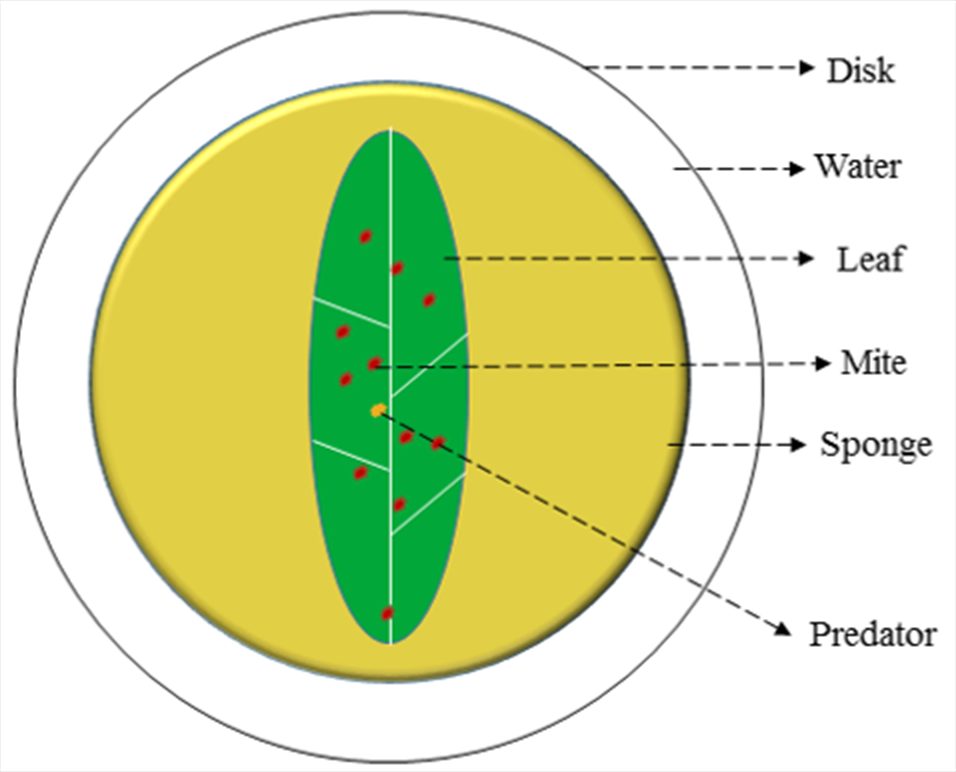

Supplement: Supplementary file 2 [file Presentation2.ZIP › Supplemetary Figures/Figure S5.tif]

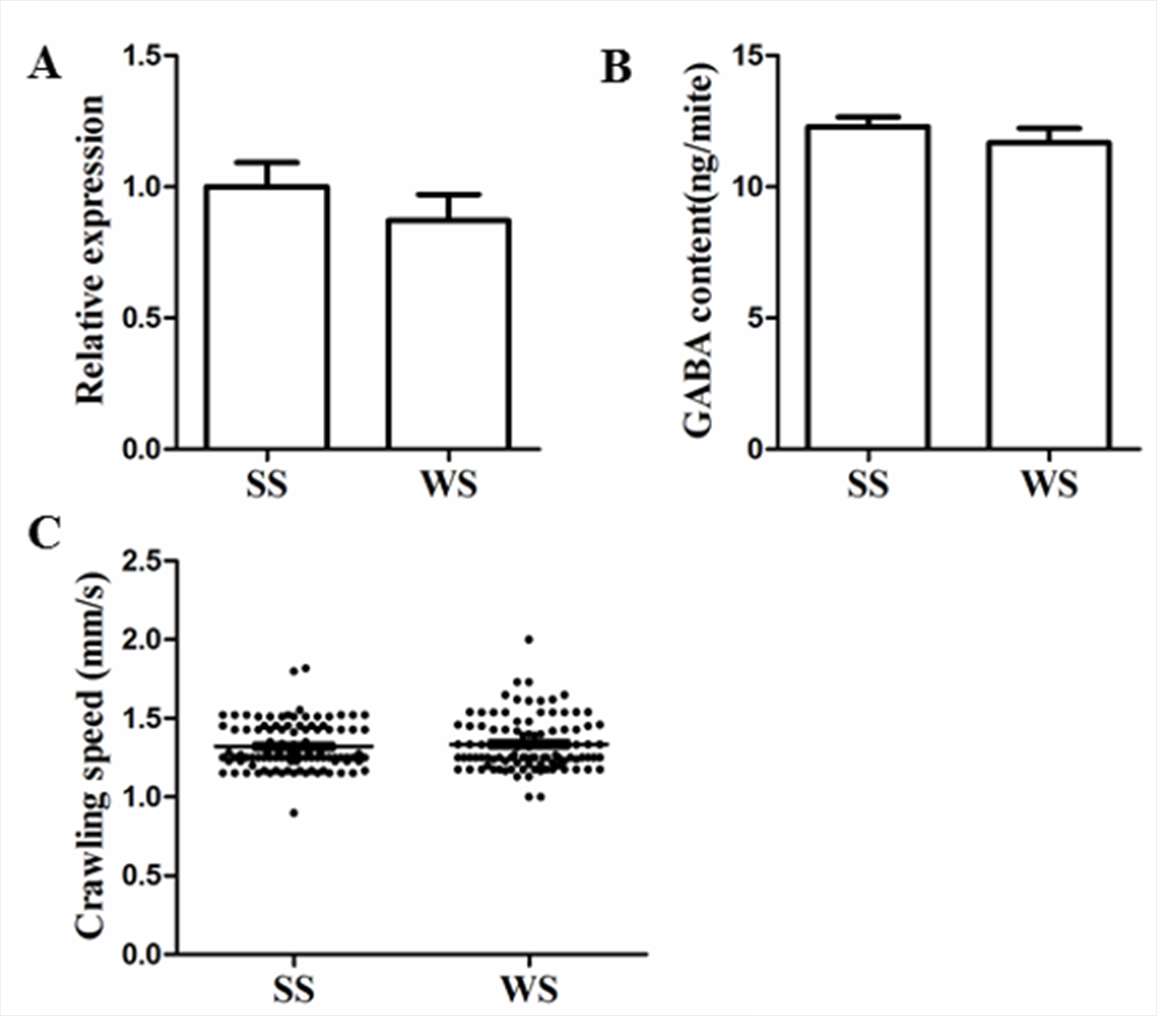

Supplement: Supplementary file 2 [file Presentation2.ZIP › Supplemetary Figures/Figure S6.tif]
