# Supplementary material for: High Gama-Aminobutyric Acid Contents Involved in Abamectin Resistance and Predation, an Interesting Phenomenon in Spider Mites
Source: Front Physiol. 2017 Apr 11;8:216. doi: 10.3389/fphys.2017.00216 (PMC5387048; doi:10.3389/fphys.2017.00216)
Supplement: Supplementary file 1 [file Presentation1.ZIP › Supplementary Material.doc]

Supplementary Material

**High gama-aminobutyric acid contents involved in abamectin resistance and predation, an interesting phenomenon in arthropods**

**Zhifeng Xu^1^, Yanchao Liu^1^, Peng Wei^1^, Jinzhi Niu^1^, Guangmao Shen^1^, Wencai Lu^1^, Wei Xiao^1^, Jinjun Wang^1^,** **Guy J. Smagghe^1, 2*^, Qiang Xu^3*^ and Lin He^1*^**

* **Correspondence:**
Corresponding Author

Qiang Xu, qxx07a@acu.edu

Lin He, helinok@vip.tom.com

**1 Supplementary Material**

**1.1 Gene cloning**

Total RNA was extracted from 200 females with TRNzol Reagent (Tiangen, Beijing, China) according to manufacturer’s instructions, and cDNA was synthesized using the Primerscript RT reagent kit (TakaRa, Dalian, China). Specific primers (Table S1) were designed based on the transcriptome data of *T. cinnabarinus*. PCR amplification was conducted in a 50 μL mixture containing 1 μL of cDNA, 5μL 10x PCR buffer, 0.2 μM of each gene-specific primers, 200 μM of each dNTP, 1.5 mM MgCl_2_, and 2.5 U Taq polymerase (TakaRa, Dalian, China). PCR was started by an initial denaturation at 94°C for 3 min, followed by 35 cycles (30 s at 94°C, 30 s at 45-58°C for gene-specific primers, and 0.5-1.5 min at 72°C) of amplification, and a final cycle of 10 min at 72°C.

5’-RACE and 3’-RACE first strand cDNAs were synthesized and PCR reaction system was constructed according to the instructions of Smart Race cDNA Amplification Kit (TakaRa, Dalian, China). According to the sequence of the PCR fragment, four genes specific primers (Table 1) were designed to amplify the full length of four genes cDNA. High fidelity DNA Polymerase (Prime START HS DNA polymerase, Takara) was used to ensure efficient and accurate amplification. The PCR amplification was as described above.

The products of PCR were analyzed on a 1% agarose gel. Purified cDNAs fragments were cloned into the pGEM-T easy vector, and then sequenced by BGI, China.

**1.2 Sequence and data analysis.** cDNA sequences and derived amino acid sequences were analyzed using the DNAstar program. Sequences similarity/annotations and analysis for conserved domains were performed using BLAST programs on the National Center for Biotechnology Information (NCBI) and the *T. urticae* genome (http://bioinformatics.psb.ugent.be/webtools/bogas). Two phylogenetic trees were constructed using the programs of MEGA 5.05 based on the amino acid sequences of GABA-T and GAD using the bootstrapping N-J tree method (algorism: Poisson correction, bootstrap values: 1000 replicates). Statistical difference was determined by One-way ANOVA followed the Tukey's post hoc test and paired samples t-test.

**Figure S1.** Phylogenetic tree of *T. cinnabarinus* GAD^TCI^ and GAD-T^TC2^ genes with other GAD genes from mammalians and arthropods. For accession numbers see S3 Table.

**Figure S2.** Phylogenetic tree of *T. cinnabarinus* GABA-T^TCI^ and GABA-T^TC2^ genes with other GABA-T genes from mammalians and arthropods. For accession numbers see S3 Table.

**Figure S3.** Inhibition of GABA-T activity by vigabatrin in SS. Error bars indicate SE (n =3). Asterisk indicates a significant difference (P<0.05).

**Figure S4.** The relative mRNA expression of GABA-T gene in SS via RNAi. The significant difference (P<0.05) was indicated by letters.

**Figure S5.** The device was used for attack ability measurement of predatory mites.

**Figure S6.** Comparison of GABA-T level, GABA content and crawling speed between SS and WS strains. A. The mRNA expression of GABA-T, SS strain as 1. B. GABA content. C. Crawling speed. Letters indicate a significant difference (P<0.05).

**Table S1. List of primers for the cloning of the four genes from CSM.**

| Gene | Name | Oligonucleotide sequence（5’ to 3’） |
| --- | --- | --- |
| GABA-T^TC1^ | GABA-T^TC1^-F1 | AGCAACAGCGACAAATGA |
|  | GABA-T^TC1^-R1 | TATCTTCTAATGGGTATCG |
|  | GABA-T^TC1^-F2 | ATGGCTTGTGGTTCTTGT |
|  | GABA-T^TC1^-R2 | GTGATACTAAGCCAGGGA |
|  | GABA-T^TC1^5RACE Outer | GTGGCTTTGTTTTCACGCTCATTAT |
|  | GABA-T^TC1^5RACE Inner | AACTGAACAAGAACCACAAGCCAT |
|  | GABA-T^TC1^3RACE Outer | AGATAATGAGCGTGAAAACAAAGCC |
|  | GABA-T^TC1^3RACE Inner | TGCTGACAGGTGGTTATTTCTACAA |
| GABA-T^TC2^ | GABA-T^TC2^-F1 | GCTTCTTTACTTTCAGTCGC |
|  | GABA-T^TC2^-R1 | GACGACCTTTTCTATTGAAT |
|  | GABA-T^TC2^-F2 | AACAAAGCCACAGATGAC |
|  | GABA-T^TC2^R2 | TCAGCTATTGTTCAGCTC |
|  | GABA-T^TC2^5RACE Outer | CAATGTTCGTGAGCCCAAAACTTAC |
|  | GABA-T^TC2^5RACE Inner | GACGACCTTTTCTATTGAATCTCTC |
|  | GABA-T^TC2^3RACE Outer | TTCGTGTTTAGCCAACCAGTATCCT |
|  | GABA-T^TC2^3RACE Inner | GCCAAGTCTCAGCAGGTTGTTTTTA |
| GAD^TC1^ | GAD^TC1^-F1 | CCTTGCTCCAGGTGGTGCTA |
|  | GAD^TC1^-R1 | ATCCTCCACCCCAAGCAGCATC |
|  | GAD^TC1^-F2 | CATTGATGCTGCTTGGGGTG |
|  | GAD^TC1^-R2 | CTTCTCATAGCCAGTGTC |
|  | GAD^TC1^-5RACE Outer | GAACCGTTGAACACTGAAGTAGAGT |
|  | GAD^TC1^-5RACE Inner | CTCTCTCAATACCAGCCAAACGATG |
|  | GAD^TC1^-3RACE Outer | GACATCGTTTGGCTGGTATTGAGAG |
|  | GAD^TC1^-3RACE Inner | CAAAGGTGACACTGGCTATGAGAAG |
| GAD^TC2^ | GAD^TC2^-F | CGTAAAGTGGCTGAAATGGTTC |
|  | GAD^TC2^-R | ACAGGTCAAAGGCTGATAT |
|  | GAD^TC2^-5RACE Outer | ACAGATTGCTGCTACCTCTCGGATT |
|  | GAD^TC2^-5RACE Inner | AAGAACCATTTCAGCCACTTTACGA |
|  | GAD^TC2^-3RACE Outer | GAATCGTTACATAACAGAT |
|  | GAD^TC2^-3RACE Inner | ATATCAGCCTTTGACCTGT |

**Table S2. Primers used for the amplification of the quantitative real-time PCR and RNAi from *T. cinnabarinus* GABA-T and GAD.**

| Gene | Primer name | Sequences(5’ to 3’) | Ref./usage |
| --- | --- | --- | --- |
| GABA-T | GABA-T-PSF | CGTGATGGTCGTGAAGTGAC | qPCR |
|  | GABA-T-PSR | CGAATGAAGGGACATCCAAT |  |
| GAD^TC1^ | GAD^TC1^-PSF | TGCCATTATTGTTGCTCGAC | qPCR |
|  | GAD^TC1^--PSR | TCGGTGACCCTTTTCGATAG |  |
| GAD^TC2^ | GAD^TC2^-PSF | CGATCAATTTGGTGTTGCTG | qPCR |
|  | GAD^TC2^-PSR | AACCGCCTGGTGAGAAGATA |  |
| RPS18 | RPS18-F | ACTACGCTCGTGGCCACTATACAA | Sun et al. (2010) |
|  | RPS18-R | ACCAGATCCAGTTCCACCTCCAAA |  |
| GABA-T | GT-RNAi-F | TAATACGACTCACTATAGGGCGTGATGGTCGTGAAGTGAC | RNAi |
|  | GT-RNAi-R | TAATACGACTCACTATAGGGCGAATGAAGGGACATCCAAT |  |

The qPCR primer of GABA-T was used detected the expression levels of GABA-T^TC1^ and GABA-T^TC2^.

**Table S3. GenBank accession numbers of GABA-T and GAD genes used for phylogenetic analysis.**

| Species | Accession no. | Species | Accession no. |
| --- | --- | --- | --- |
| **GABA-T** |  |  |  |
| *Tetranychus cinabarinus* | JQ942361 | *Tetranychus cinabarinus* | JQ942362 |
| *Tetranychus urticae* | tetur01g01480 | *Tetranychus urticae* | tetur10g00500 |
| *Drosophila virilis* | XP_002048010 | *Bombyx mori* | XP_004924846 |
| *Metaseiulus occidentalis* | XP_003738558 | *Bombus terrestris* | XP_003397486 |
| *Ixodes scapularis* | XP_002405970 | *Apis dorsata* | XP_006614968 |
| *Daphnia pulex* | EFX76516 | *Anopheles darlingi* | ETN61729 |
| *Bombus impatiens* | XP_003492665 | *Apis mellifera* | XP_392483 |
| *Callorhinchus milii* | XP_007891964 | *Apis florea* | XP_001607115 |
| *Peromyscus maniculatus bairdii* | XP_006992185 | *Microtus ochrogaster* | XP_005349342 |
| *Musca domestica* | XP_005182496 | *Drosophila mojavensis* | XP_002009283 |
| *Ovis aries* | XP_004020808 | *Pantholops hodgsonii* | XP_005984717 |
| *Melopsittacus undulatus* | XP_005150763 | *Geospiza fortis* | XP_005420493 |
| *Lepisosteus oculatus* | XP_006637224 | *Lepisosteus oculatus* | XP_006637225 |
| *Camponotus floridanus* | EFN68249 | *Drosophila grimshawi* | XP_001985648 |
| *Anas platyrhynchos* | EOB08957 | *Anas platyrhynchos* | XP_005009196 |
| *Bos taurus* | DAA15551 | *Tarsius syrichta* | XP_008055858 |
| *Columba livia* | XP_005508492 | *Drosophila willistoni* | XP_002065822 |
| *Bubalus bubalis* | XP_006063443 | *Acromyrmex echinatior* | EGI67372 |
| *Anopheles gambiae* | XP_308791 | *Mus musculus* | BAE21080 |
| *Aedes aegypti* | XP_001656148 | *Daphnia pulex* | EFX78146 |
| *Ceratitis capitata* | XP_004523005 | *Tupaia chinensis* | XP_006140766 |
| *Cavia porcellus* | XP_003477790 | *Pelodiscus sinensis* | XP_006124654 |
| *Balaenoptera acutorostrata scammoni* | XP_007188479 | *Drosophila persimilis* | XP_002028308 |
| *Falco peregrinus* | XP_005244171 | *Harpegnathos saltator* | EFN75225 |
| *Gallus gallus* | XP_414940 | *Lipotes vexillifer* | XP_007469990 |
| *Orcinus orca* | XP_004270247 | *Mus musculus* | NP_766549 |
| *Physeter catodon* | XP_007116080 | *Salmo salar* | ACI33107 |
| *Cricetulus griseus* | XP_003496426 | *Taeniopygia guttata* | XP_002196729 |
| *Bos taurus* | NP_001075050 | *Zonotrichia albicollis* | XP_005487692 |
| *Saimiri boliviensis boliviensis* | XP_003928553 | *Pongo abelii* | NP_001127257 |
| *Pseudopodoces humilis* | XP_005522932 | *Mesocricetus auratus* | XP_005084607 |
| *Homo sapiens* | AAH31413 | *Homo sapiens* | NP_065737 |
| *Loxodonta africana* | XP_003417679 | *Heterocephalus glaber* | EHB12191 |
| *Sorex araneus* | XP_004604194 | *Salmo salar* | ACI33512 |
| *Gorilla gorilla gorilla* | XP_004057219 | *Danio rerio* | NP_958906 |
| *Rattus norvegicus* | XP_006245822 | *Homo sapiens* | 2104211A |
| *Rattus norvegicus* | NP_112265 | *Homo sapiens* | XP_005255211 |
| *Chinchilla lanigera* | XP_005391276 | *Camponotus floridanus* | EFN68248 |
| *Callithrix jacchus* | XP_002755924 | *Myotis lucifugus* | XP_006090442 |
| *Heterocephalus glaber* | XP_004899236 | *Heterocephalus glaber* | XP_004899235 |
| *Octodon degus* | XP_004627832 | *Megachile rotundata* | XP_003704536 |
| *Myotis davidii* | XP_006763649 | *Chelonia mydas* | XP_007053855 |
| *Chrysemys picta bellii* | XP_005295305 | *Tetranychus urticae* | tetur01g01480 |
| *Tetranychus urticae* | tetur10g00500 |  |  |
| **GAD** |  |  |  |
| *Tetranychus cinabarinus* | JQ942360 | *Tetranychus cinabarinus* | JQ798396 |
| *Panonychus citri* | AGT28747 | *Rhipicephalus microplus* | AFX68719 |
| *Apis dorsata* | XP_006609683 | *Apis florea* | XP_003692672 |
| *Bombus impatiens* | XP_003494604 | *Megachile rotundata* | XP_003703237 |
| *Bombus terrestris* | XP_003400061 | *Drosophila ananassae* | XP_001956770 |
| *Bombus impatiens* | XP_003494603 | *Anopheles darlingi* | ETN61760 |
| *Culex quinquefasciatus* | XP_001868526 | *Drosophila mojavensis* | XP_002009153 |
| *Camponotus floridanus* | EFN63631 | *Drosophila grimshawi* | XP_001985333 |
| *Drosophila pseudoobscura* | XP_001353656 | *Drosophila willistoni* | XP_002068379 |
| *Metaseiulus occidentalis* | XP_003743720 | *Acromyrmex echinatior* | EGI60033 |
| *Drosophila melanogaster* | NP_523914 | *Harpegnathos saltator* | EFN79405 |
| *Bombyx mori* | XP_004925034 | *Drosophila virilis* | XP_002047764 |
| *Cerapachys biroi* | EZA52347 | *Ceratitis capitata* | XP_004525301 |
| *Zonotrichia albicollis* | XP_005484106 | *Geospiza fortis* | XP_005415906 |
| *Drosophila simulans* | XP_002083572 | *Rattus norvegicus* | 1713398A |
| *Drosophila melanogaster* | CAA53791 | *Elephantulus edwardii* | XP_006878914 |
| *Chrysochloris asiatica* | XP_006866680 | *Orycteropus afer* | XP_007942576 |
| *Anas platyrhynchos* | XP_005018985 | *Falco peregrinus* | XP_005237155 |
| *Camelus ferus* | EPY77921 | *Tetranychus urticae* | tetur19g02930 |
| *Tetranychus urticae* | tetur22g02360 |  |  |

*Tetranychus urticae* accession numbers can be accessed through the BOGAS genome portal (http://bioinformatics.psb.ugent.be/webtools/bogas/), other accession numbers are available at GenBank. NCBI BLASTp hits (*E*-value<10^-5^).
